# Automatic extraction of ranked SNP-phenotype associations from text using a BERT-LSTM-based method

**DOI:** 10.1186/s12859-023-05236-w

**Published:** 2023-04-12

**Authors:** Behrouz Bokharaeian, Mohammad Dehghani, Alberto Diaz

**Affiliations:** 1 Amol University of Special Modern Technologies, Mazandaran, Iran; 2 School of Electrical and Computer Engineering, University of Tehran, Tehran, Iran; 3 Facultad Informatica, Complutense University of Madrid, Madrid, Spain

**Keywords:** SNP, Phenotype, Biomedical relation extraction, Degree of certainty classification

## Abstract

Extraction of associations of singular nucleotide polymorphism (SNP) and phenotypes from biomedical literature is a vital task in BioNLP. Recently, some methods have been developed to extract mutation-diseases affiliations. However, no accessible method of extracting associations of SNP-phenotype from content considers their degree of certainty. In this paper, several machine learning methods were developed to extract ranked SNP-phenotype associations from biomedical abstracts and then were compared to each other. In addition, shallow machine learning methods, including random forest, logistic regression, and decision tree and two kernel-based methods like subtree and local context, a rule-based and a deep CNN-LSTM-based and two BERT-based methods were developed in this study to extract associations. Furthermore, the experiments indicated that although the used linguist features could be employed to implement a superior association extraction method outperforming the kernel-based counterparts, the used deep learning and BERT-based methods exhibited the best performance. However, the used PubMedBERT-LSTM outperformed the other developed methods among the used methods. Moreover, similar experiments were conducted to estimate the degree of certainty of the extracted association, which can be used to assess the strength of the reported association. The experiments revealed that our proposed PubMedBERT–CNN-LSTM method outperformed the sophisticated methods on the task.

## Introduction

**Fig. 1:**
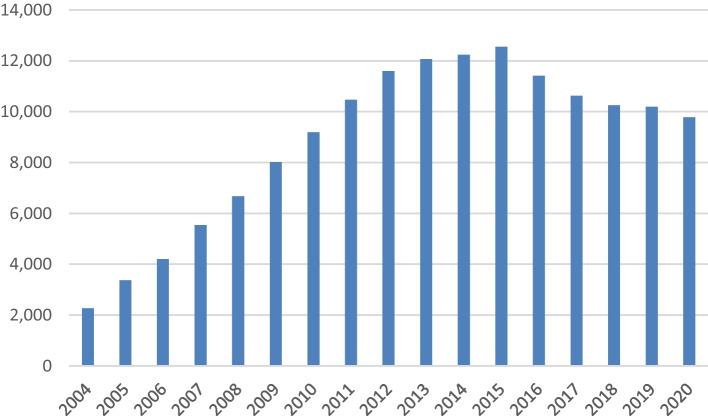
Number of “Single Nucleotide Polymorphisms” related publications from 2004 to 2020 in the PubMed

A single-nucleotide polymorphism (SNP) is a single-base mutation at the DNA level [1]. Variations in the DNA sequences can affect how humans develop diseases and respond to pathogens, chemicals, drugs, and other agents. A genome-wide association (GWA) study is an observational study of a set of genome-wide genetic variations in different individuals to determine if the mutation is associated with a trait like a major human disease. The first successful GWA study dates back to 2005, when Klein et al. performed the first successful GWAS on patients with age-related macular degeneration. It was the beginning of a worldwide trend, finding thousands of SNP associations. Figure 1 depicts the increasing number of papers published in the field from 2004 to 2020, which were obtained from a PubMed search engine for the query “Single Nucleotide Polymorphisms” (performed in November 2021). SNPs are also crucial for personalized medicine.

A phenotype is an organism's recognizable characteristics or traits such as its development, biochemical or physiological properties, behavior, and products of behavior [2]. An SNP can "relate" to a phenotype when a specific type of variant (one allele) is frequent within samples obtained from subjects. The degree to which genotype determines phenotype is referred to as "phenotypic plasticity" [3].

There are genetic instructions for growing and developing all individuals; however, environmental parameters influence an individual’s phenotype through embryonic growth and life. The amount of influence that environmental factors have on an individual’s ultimate phenotype is a serious scientific debate. Environmental parameters can result from various effects, including nutrition, weather, disease, and stress level. For example, the ability to taste the food is a phenotype estimated as 85% affected via genetic inheritance [4]. Additionally, the ability could be intervened by environmental parameters such as dry mouth and lately eaten food. However, phenotypic plasticity is considered high if environmental factors have a strong influence. Conversely, if phenotypic plasticity is low, the genotype can be used to predict phenotypes reliably. Overall, the amount of the influence of environmental factors on a phenotype is a source of scientific arguments. However, the large amount of data generated from these studies necessitates developing an automatic approach to facilitate the study of extracted associations.

**Fig. 2:**
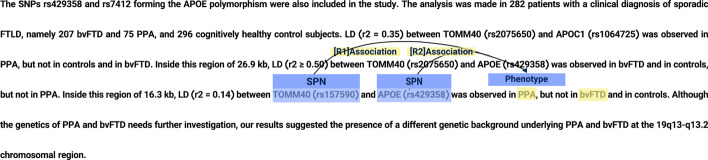
Sample of SNP-Phenotype associations

Recently, few methods have been developed to extract mutation and disease associations from text such as [5] and [6]. Owing to the importance of the task, the authors produced the SNPPhenA corpus that can be used for benchmarking purposes [7]. Figure 2 presents two sample associations between two SNPs highlighted with blue and a Phenotype (PPA) highlighted with green.

The procedure of producing the corpus consisted of gathering the related abstract and named-entity recognition and annotating the associations, negation, modality markers, and degree of certainty for associations.

Identification of negations in the text is one of the essential tasks in biomedical text mining. Linguists define negation as a morphosyntactic operation [8], and a lexical item either denies or inverts the meaning of another item or construction through this operation. The importance of negation in biomedical text mining is revealed when we consider the negation commonplace in those texts, leading to a lack of precision in automatic information retrieval systems [9]. For example, in the sentence below, there is not no association between "*APOE polymorphisms*" and "*serum HDL-C*"; however, if the negation is neglected, a wrong association might be identified:

- There were < { no} associations between *APOE polymorphisms* and *serum HDL-C*, APO-CIII, and triglycerides > 

Linguistic modality is another linguistically driven phenomenon to be applied in this research. In general, modals are particular words that state modality and express the announcer’s internal attitudes and beliefs such as facility, probability, inevitability, commitment, permissibility, capability, wish, and contingency [10]. In the current study, the author’s confidence in the sentence is determined to show the strength of the SNP-phenotype associations stated in the corpus.

Although many machine learning methods have been used to extract biomedical relations from text, recent advances in biomedical text mining techniques have occurred through deep learning models [11, 12]. Nevertheless, direct use of sophisticated natural language processing (NLP) methodologies to extract biomedical relations have some limitations. However, the biomedical text mining model may often encounter problems of general corpora. Therefore, recent biomedical text mining models rely primarily on the adapted versions of word representations such as SciBERT [13] for scientific texts and PubMedBERT-LSTM [14] for biomedical texts.

In this study, the authors develop and compare some common machine learning techniques, along with some deep learning-based approaches that extract associations between SNPs and phenotypes. The rest of this paper is organized as follows. Section "SNPPhenA Corpus" discusses some of the fundamental characteristics of the SNPPhenA corpus, and section "Related works" introduces some related research works. Section "Method" expounds the proposed methods. Afterward, section "Evaluation" presents the results and statistical analysis. Finally, section "Discussion and conclusion" concludes the paper and provides some suggestions for further research.

## SNPPhenA corpus

The SNPPhenA corpus was developed to extract the ranked associations of SNPs and phenotypes from GWA studies. The process of producing the corpus entailed collecting relevant abstracts and named entity recognition, and annotating the associations, negation cues and scopes, modality markers, and degree of certainty of the associations [7].

As opposed to the previous biomedical relation extraction corpora containing true and false types of relations, the associations annotated in the corpus were divided into three classes: positive, negative, and neutral candidates.

Unlike distinguished association candidates, including the author’s remarks, a neutral candidate does not contain any remarks [15]. In other words, neutral candidates were those SNP-phenotype candidates that showed no clear evidence as to the presence or lack of an association between SNPs and phenotypes. Identification of neutral candidates is critical for the negation process as the status of such candidates and their corresponding degree of certainty classification do not change when they are located in the scope of negation terms; on the contrary, the status of distinguished association candidates changes in such cases. McDonald et al. are one of the very few groups of researchers who have investigated neutral candidates in terms of the RE task [16].

Similarly, a neutral candidate's degree of certainty or uncertainty does not change if it is located in the scope of a speculation or modality term. Hence, determination of the effect of negation as well as modality terms requires identification of neutral candidates.

### Examples

**Fig. 3:**
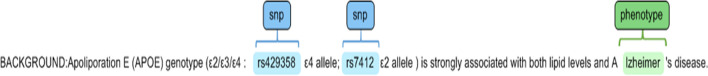
Samples of a positive association between the two highlighted SNPs and a phenotype

**Fig. 4:**
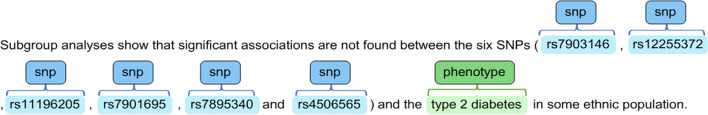
Samples of a negative association between the six highlighted SNPs and a phenotype

**Fig. 5:**
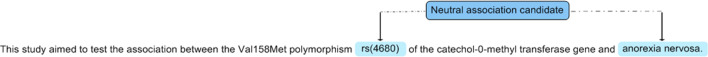
A sample of a neutral association with the employed highlighted entities

**Fig. 6:**
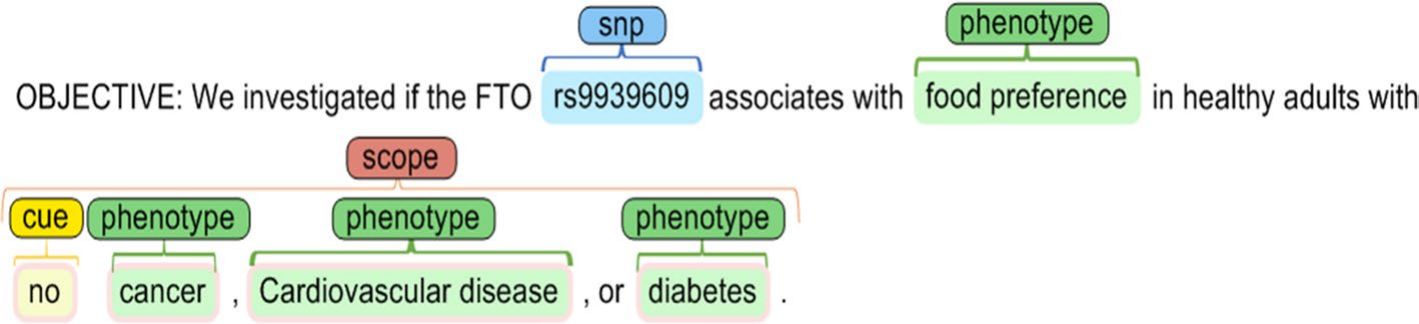
A sample of a neutral association with a negation cue

SNP-phenotype candidates were classified as positive, negative, and neutral. Positive SNP-phenotype relation candidates are those with clearly indicated associations (Fig. 3). In contrast, negative SNP-phenotype relation candidates are those in which a lack of association is evident (Fig. 4). In addition to the typical classes of relationships, a neutral class is defined for those within the two other classes, where the presence or absence of association is not noted in the sentence (see Figs. 5 and 6).

**Fig. 7:**
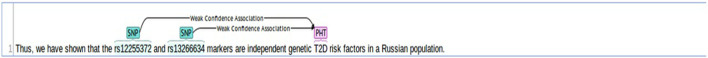
A sample of a weak association deemed to have a weak degree of certainty

**Fig. 8:**
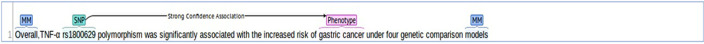
A sample of a strong association considered to have a strong degree of certainty

In addition to the mentioned annotations, the confident level of a positive association in the corpus was annotated in three categories: strong, moderate, and weak degree of certainty. Figures 7 and 8 display two samples of weak and strong associations.

### Characteristics of the SNPPhenA corpus

This section provides detailed statistics regarding the linguistic and non-linguistic properties of the corpus. Table 1 presents the basic properties of the corpus, including the statistics of the produced corpus in terms of test and training parts. As the table shows, the candidates with a positive association comprised the largest category, while the negatively associated candidates constituted the smallest category.

**Table 1:** Basic statistics of the SNPPhenA corpus in terms of test and train parts

Item	Train	Test	Total
Files	270	90	360
Abstracts	270	90	360
Sentences	1940	685	2525
Key sentences	463	156	619
SNP	632	236	868
Phenotypes	445	145	590
SNP-phenotype candidates	786	342	1128
Neutral candidates	77	103	180
Negative candidates	86	239	325
Positive candidates	623	188	811
Files	270	90	360
Abstracts	270	90	360
Sentences	1940	685	2525
Key sentences	463	156	619
SNP	632	236	868
Phenotypes	445	145	590
SNP-phenotype candidates	786	342	1128
Neutral candidates	77	103	180
Negative candidates	86	239	325
Positive candidates	623	188	811

Table 2 provides detailed analyses concerning the different types of SNP-phenotype association candidates. As mentioned, the key negated sentences in the corpus were annotated with scopes of negation and negation cues. As Table 2 shows, 18.5% of the sentences had at least one negation cue. Further analysis shows that "not" and "no", with respective occurrences of 35 and 38, were the most frequent negation cues. According to the conducted analyses, each sentence in the corpus had an average of 76.9 tokens, 1.7 SNPs, and 1.2 phenotypes.

**Table 2:** Statistics of different types of SNP-phenotype association candidates in the SNPPhenA corpus

Item	Number	Percentage (%)
Total SNP-phenotype association candidates	1128	100
Candidate with at least a negation cue	218	18.5
Candidates with only one negation cue	189	16.3
Candidates with clause connectors	823	63.8
Candidates without clause connector	470	36.2
Weak degree of certainty positive candidates	515	39.6
Normal degree of certainty positive candidates	124	9.5
High degree of certainty positive candidates	233	17.9

As Table 1 illustrates, 76.3% of the samples are distinguished (i.e., they are positive and negative association candidates). Therefore, it can be concluded that the annotated sentences were expressed primarily as a direct mechanism or association between one or more SNPs and a phenotype.

As Table 2 shows, 63.8% of the candidate sentences had at least one clause connector, while 36.2% did not have it. The statistical analysis on the clause connectors further indicated that 9.7% (= 87/895) of instances had concessive clauses.

Additionally, as Table 2 shows, 63.8% of the candidate sentences had at least one clause connector, while 36.2% did not have it. The statistical analysis on the clause connectors further indicated that 9.7% (= 87/895) of instances had concessive clauses.

Tables 3 and 4 present the most frequent phenotypes, SNPs, and some basic statistics concerning the produced corpus.

**Table 3:** Some of the most occurred phenotypes in the corpus

Phenotype/ phenotypic phenotype	Num. of abstracts
Health risk	40
Smoking	33
Obesity	25
Metabolic syndrome	16
Hypertension	10
Insulin sensitivity	9
Hypertriglyceridemia	7
Glucose metabolism	6
Impaired glucose tolerance	5
Longevity	4
Body mass intake	4
Cognitive performance	4
Skin pigmentation	3
AIDS	3

**Table 4:** SNPs with the highest occurrence in the SNPPhenA corpus

SNP	Number of abstracts
rs12255372	78
rs429358	55
rs7412	46
rs4680	38
rs1051730	25
rs662799	20
rs1799971	18
rs1800629	14

Moreover, the inter-annotator agreement was analyzed, the Kappa coefficient was calculated for SNP-phenotype associations, and the degree of certainty of associations demonstrated the reliability of the corpus. The Kappa inter-annotator agreement between the two annotators was 0.79 for annotating the associations and 0.80 for annotating the degree of certainty of associations, demonstrating the reliability of the corpus.

## Related works

In addition to classical relation extraction tasks in the BioNLP domain, such as protein–protein and gene-disease tasks, some new methods and corpora have been developed to extract mutation/polymorphism and disease associations. DiMex [6] is a rule-based unsupervised mutation-disease association extraction working at the abstract level. The PKDE4J [5] is a supervised method employing a rich set of rules to detect the used features. Another related miner system has been presented [17] that gathers heterogeneous data from various literature sources to draw new inferences regarding the target protein families.

Similar studies using standard machine learning methods on this task have yielded significant results [18, 19]. A similar work has been presented by [20] that used finite state automata and random forest-weighted.

More recent works have been presented by [21, 22], and [23], in which deep learning and pre-trained models were employed to extract gene mutation-disease relations from the literature. [24] provides an interesting review of new methods for extracting the genomic variant information from the literature.

Among the other similar tasks, Asada et al. proposed a new BERT and CNN-based method to extract DDIs from text using drug descriptions and molecular structures that outperformed other approaches [25]. Liu et al. conducted similar research on the DDI extraction from the literature. They proposed a TM-RNN method by adding the transfer weight matrix in a multilayer bidirectional LSTM to introduce a memory network for feature fusion [26]. In addition to previous works, Legrand et al. employed a transfer learning method called TreeLSTM with biomedical domain adaptation. They also demonstrated the crucial role of syntax in transfer learning [27]. Biotian et al. [28] reported another similar study. They revealed that open-domain reading comprehension data and knowledge representation could help to improve biomedical relation extraction.

Another interesting research used the features obtained from both the BEST search engine scores and word vectors, along with a deep convolutional neural network to extract mutation-gene and mutation-drug relations from text. It can be used to identify molecular biomarkers predicting drug responses in cancer patients [27].

In addition to the mentioned methods, some researchers explored the use of linguistic features like negation and speculation phenomena separately for this task. [29] was one of the few pieces of research that considered negation in relation to extraction tasks. In this method, the SVM classifier was fed using a list of features such as the nearest verb to candidate entity in the parse tree and some negation cues. Pyysalo et al. [30] conducted one survey in which negation and uncertainty issues were considered. They stated that among the corpora, BioInfer had negative annotation. Numerous studies have investigated the modality and speculation of identification in NLP [31]; however, only few studies have been employed to classify the speculative language under bioscience texts.

## Method

In this research, several experiments were conducted using different families of classifiers, including kernel-based, semantically linguistic-based, random forest, and deep learning-based methods to extract SNP-phenotype associations and their degrees of certainty.

In this section, the authors initially explain the methods developed for extracting the SNP-phenotype associations, and then they describe the techniques developed for extracting the degree of certainty of the associations. Different phenotypic plasticity, as well as other effective unknown genetic components, presents two explanations for the GWA study reports on the importance of the degree of certainty for the associations. Consequently, the linguist-based degree of certainty of the reported associations will have informative data to determine phenotypic plasticity. However, there is no available automatic method for extracting the degree of certainty of the results. Consequently, the presence of such a tool and data source is critical and can be applied to help researchers to review the literature.

It is worth mentioning that NLTK and spacy were used for preprocessing, and scikit-learn was employed to implement the machine learning methods. In addition, experiments based on the developed deep learning models were conducted by PyTorch, TensorFlow, and Keras libraries. The BERT-based related experiments were performed by Transformers and Torch libraries. All kernel methods experiments were conducted by a support vector with an implementation of SMO [32]. According to the experiments conducted via the SMO approach and comparing the results to those of other implementations of SVM, e.g., libSVM, it was evident that the SMO implementation was a better option as it performed faster. It is worth mentioning that we used the SNPPhenA corpus during the research which was introduced previously [7]. The corpus is available for public use [1].

### Extracting ranked SNP-phenotype associations from text using machine learning methods

In this research, we conducted several experiments for the ranked association extraction using several machine learning and two kernel-based methods that have been proved to be popular among researchers. Initially, the authors explain the methods used to extract the associations and then elaborate on the other subtasks, which is the classification of the degree of certainty of the associations.

#### Extracting SNP-phenotype associations

The used kernel methods are tree kernel, local context, and subtree kernels, which have been used in several studies and have exhibited good performance, particularly in combination with other kernels [33]. Furthermore, random forest, logistic regression, decision tree, GradientBoosting, GaussianNB, and KNN are applied for classifying the associations.

A grid-search algorithm was used to optimize the performance of the algorithms. For example, the logistic regression classifier's parameter space algorithm resulted in the following parameters: C = 100, penalty = 1.0, solver = 5. For this purpose, the used grid-search algorithm examined seven numbers for the gamma parameter, which were between -3 and + 3, and the best penalty parameter was selected from l1 and l2 values, and the solver parameter was selected from liblinear and newton-cg methods.

Additionally, we used a weighting method based on a categorical cross-entropy loss function for dealing with imbalanced data in the SNPPhenA corpus. We assigned the weights based on the ratio of the number of samples in each class. More details of such a weighting method can be found in [34].

The evaluation section describes the results of the used SNP-Phenotype association extraction methods.

#### Degree of certainty classification

The authors conducted several experiments using shallow machine learning and kernel methods in this research. The machine learning methods include LogisticRegression, RandomForest, KNN, GaussianNB, DecisionTree, and GradientBoosting; all of the commonly used classifiers identify three degrees of certainty of associations after preprocessing and tokenization steps. The implemented machine learning algorithms had to be optimized, since two different tasks existed. To improve the results, similar to the association extraction subtask, the machine learning algorithms were optimized using a grid search algorithm to fine-tune the models. For instance, employing the fine-tuning of the parameters of the random-forest method using grid-search led to criterion = 'entropy', min_samples_split = 3, min_samples_leaf = 2.

### Semantically linguistic-based ranked SNP-phenotype association extraction

This section provides the details of the proposed method. The proposed association extraction method relies on detecting linguistic-based negation and neutral candidates introduced in this section.

#### Extracting SNP-Phenotype associations using negation and neutral candidates (NNB)

**Fig. 9:**
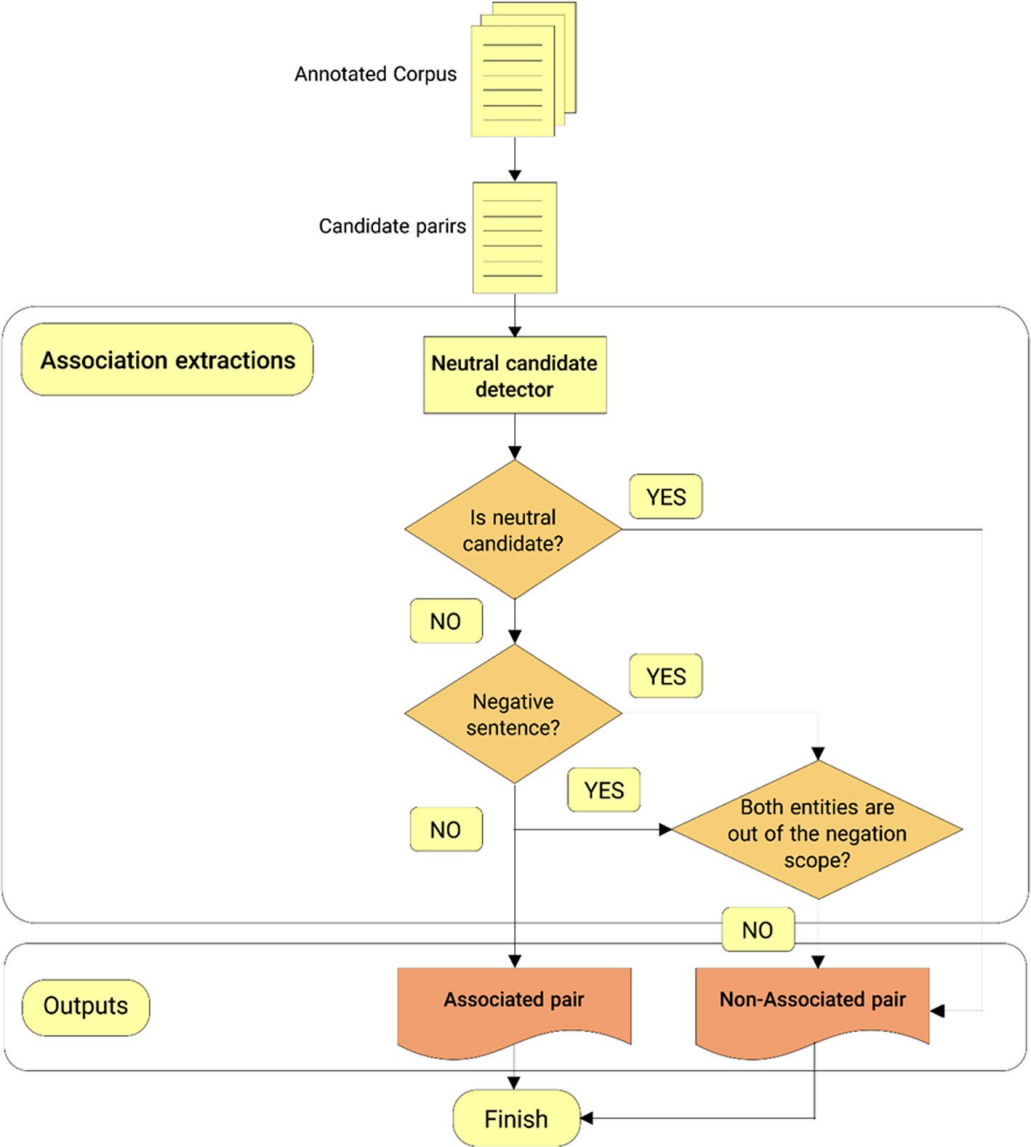
Different steps of the semantically linguistic-based SNP-phenotype association extraction proposed algorithm

Six Boolean features were extracted from negation cues and the used scope to develop the proposed approach. Additionally, neutral examples were identified in the corpus to determine possible effects of negation on the SNP-Phenotypes relation. Negation inverts the status of positive or negative relations candidates in the negation scope while leaving neutral ones unchanged. Consequently, the ratio of neutral candidates to positive or negative ones is exceedingly significant. The flowchart in Fig. 9 presents the basic components of the algorithm.

##### Neutral candidate detector

As the initial experiments show, detection of neutral candidates is vital in the negation-based method. Consequently, a neutral candidate detection system was implemented. The proposed method is a rule-based method that uses a regular expression technique similar to the authors' previous work for DDI extraction [35]. The SNPPhenA corpus train part was used to identify the produced neutral candidate rules.

Therefore, if the status of the existence of a neutral candidate was defined as:

- "IsNeutralCand" A Boolean feature, which is set as true when the association candidate is predicted as neutral, while in other situations, it is false.

##### Negation-based association extraction method

As for relation extraction, it must be noticed that negation does not necessarily change the status of a relationship between entities. Indeed, the effect of negation on association depends on several factors, among which the position of entities relative to the negation scope and cue can be directly extracted from an extended corpus. For example, consider the following sentence:

- Moreover, the ***rs1051730*** variant may ***not*** merely operate as a marker for ***dependence or heaviness of smoking***

"Dependence or heaviness of smoking" is a phenotype name inside the negation scope, so that the association relation between SNP (rs1051730) and the phenotype name is inverted by the negation. There are 6 different possibilities based on the position of SNP and phenotype names relative to the negation scope, which are used as 6 features:

- BothInsNegSc: A Boolean feature, which would be set as true when both SNP and phenotype names are inside the negation scope, while other cases are false.
- OneLeftOneInsNegSc: A Boolean feature that would be set as true when one SNP or phenotype name is on the left side (out) of the negation scope, and the other is inside the negation scope. It would be considered false in any other case.
- OneRightOneInsNegSc: A Boolean feature, which would be set as true when SNP or phenotype name is on the right side (out) of the negation scope, and the other one is inside the negation scope, while other cases are false.
- Three other Boolean features related to other possibilities.

As Fig. 9 depicts, if the studied candidate is not neutral, and one of these three Boolean features (BothinsideNegSc, OneLeftOneInsideNegSc, or OneRightOneInsideNegSc) is true, the test association is predicted as false; otherwise, any other combination of features leads to a true association.

However, In the case of neutral candidates, negation does not change the status of the association, and it will remain false. As the next section reveals, owing to the few neutral candidates in the produced corpus, consideration of neutral candidates as negatives still leads to superior performance.

The status of an association can be calculated as follows:

- $$SNPTraitAssociation = \left( {{\text{BothInsNegSc }} \vee {\text{OneLeftOneInsNegSc}} \vee {\text{OneRightOneInsideNegSc}}} \right) \wedge \neg {\text{ IsNeutralCand }}\neg$$

In the next section, the results obtained by the proposed method as well as those given by other machine learning and deep learning-based methods are presented, so that they can be easily compared to each other.

#### Degree of certainty classification

Additionally, a modality-based supervised learning method (MBS) was implemented to identify the degree of certainty of the extracted association. The proposed method consisted of an SVM classifier initially trained by the modal markers annotated in the training part of the corpus. The mentioned p-value of the sentence and the clause connector of the annotated sentences were employed as extracted features. The mentioned feature extraction phase was carried out by regular expressions [35] as well as annotations available in the corpus. Then, the modal markers, the container clause, and the extracted p-value were identified from the candidate sentence during the test phase. Ultimately, the degree of certainty was predicted to employ the trained model.

### Deep learning-based ranked SNP-phenotype association extraction models

In addition to the mentioned machine learning and the proposed rule-based methods, some experiments are conducted with three deep learning-based methods (BERT-LSTM-, PubMedBERT-LSTM-, and CNN-LSTM-based methods). As mentioned earlier, to deal with imbalanced data in the SNPPhenA corpus, we employed a weighting method based on a cross-entropy loss function.

#### Extracting SNP-phenotype associations

It has been demonstrated that pre-training large neural language models like BERT can lead to impressive performance improvements on numerous NLP tasks. Recent research indicates that pre-training language models from scratch in domains with large unlabeled text, including biomedicine, result in substantial improvements over continuous pre-training of general-domain language models.

A pre-trained PubMedBERT was trained by looking at abstracts from PubMed and full-text articles from PubMedCentral. Currently, this model holds the top score on the Biomedical Language Understanding and Reasoning Benchmark, an assessment of the NLP performance on biomedical tasks.

**Fig. 10:**
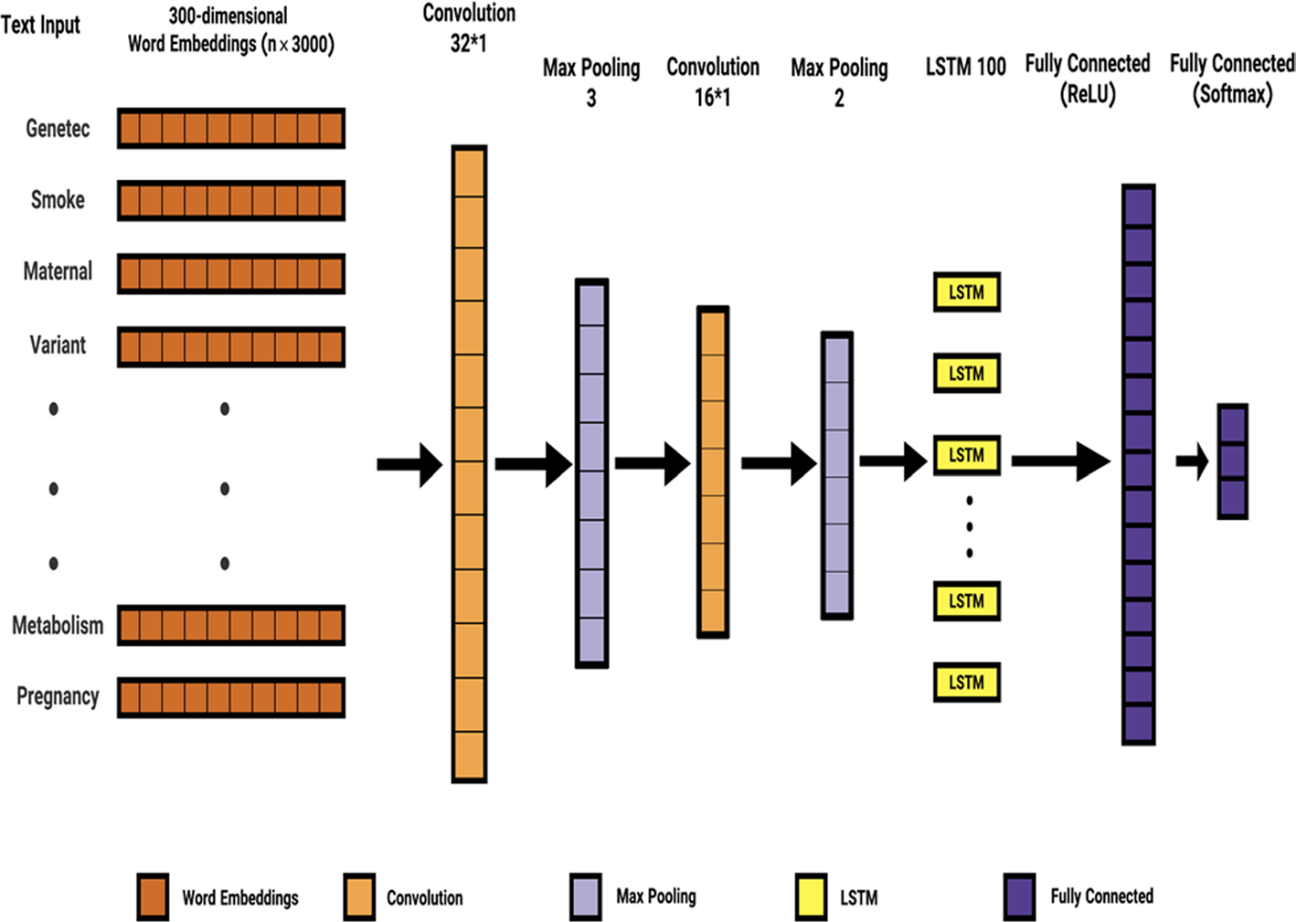
Systems architecture of the used Deep CNN–LSTM-based method

The authors adopted a deep CNN-LSTM based neural network model that exhibited acceptable performance in all the experiments. Figure 10 depicts the diagram of the used neural network.

LSTM and CNN are deep learning layers that do not require manual feature engineering and automatically learn new features.

In the next step, the authors created two consecutive CNN blocks comprising convolutional, max pooling, and LSTM. The goal was to combine CNN and LSTM in this network, as the experiments revealed that CNN could facilitate the extraction of more important features. Furthermore, LSTM was justified, since the texts were sequential. Afterward, a fully connected layer was used, and the Dense and Softmax Activator functions were applied to the last layer to predict the results correctly.

**Fig. 11:**
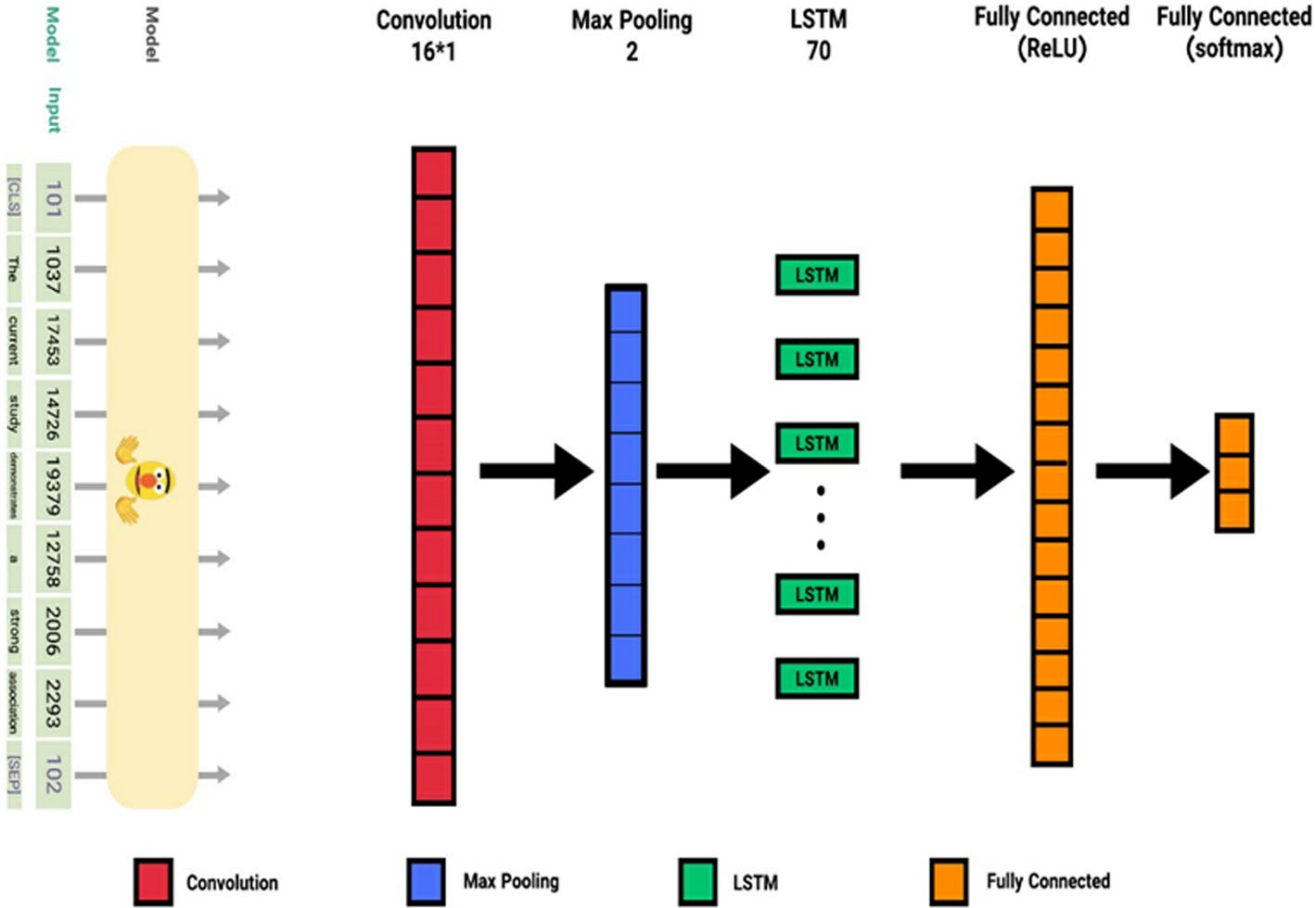
Systems architecture of the used BERT–LSTM based method

To improve the performance of the deep neural network, we added the BERT language model to the proposed system (Fig. 11). The transformer-based model employed BERT to construct token representations based on the input using multiple attention heads to attend to all tokens. Transforms do not embed positional information as they do in recurrent models; however, they still embody positional information in modeling sentence order. Early stopping is a regularization technique to prevent over fitting when learning something iteratively. When the training model is extremely short, the train and test sets will be under fitting. In the case of excessive training, the model will overfit the training dataset and perform poorly on the test set. Early stopping is a method allowing us to specify an arbitrarily large number of training.

#### Degree of certainty classification

Regarding the classification of the degree of confidence of the associations, machine learning, BERT-LSTM, PubMedBERT-LSTM, and some deep learning-based methods are used.

The core architecture of the used deep CNN-LSTM learning model is similar to that of the model used for the association extraction method (Fig. 10). However, some changes are made to suit the task, resulting in performance improvement and over fitting avoidance.

## Evaluation

In this section, after presenting some statistical analysis regarding the number of negation cues and clause connectors in the corpus, the results of the used and developed methods are presented. The training of the machine learning and deep learning methods was carried out using a training corpus, and the performance of the models was measured using the test part of the corpus. We divided the train corpus into validation and train parts. We also used the grid search method for machine learning algorithms, used the kerastuner for deep learning methods to obtain the best parameters of the model, and fine-tuned the models. In addition, we conducted some experiments using the k-fold cross validation method. The best obtained parameters and the fine-tuned models during the previous step were used for the k-fold validation method.

### Identification of SNP-phenotype associations

This section presents the comparative results of shallow machine learning, the proposed rule-based method, and deep learning-based techniques in terms of F-score.

As Tables 5 and 6 show, the experiments were conducted on two groups of candidates. During the experiments whose results are shown in Table 5, neutral candidates were considered part of the negative class of candidates as other relation extraction corpora. Tables 7 and 8 present the obtained k-fold results for the task.

**Table 5:** Comparative results at the sentence level for the identifying SNP-Phenotype associations for the test corpus with non-neutral candidates (positive and negative-neutral classes)

Model name	Precision	Recall	F1
LogisticRegression	0.770	0.63	0.59
RandomForest	0.757	0.49	0.348
KNN	0.690	0.69	0.689
GaussianNB	0.595	0.518	0.448
DecisionTree	0.761	0.638	0.604
GradientBoosting	0.72	0.581	0.524
SVM (LCK)	0.58	0.62	0.60
SVM(Subtree Kernel)	0.41	0.401	0.41
NNB	0.706	0.704	0.71
CNN-LSTM	0.732	0.723	0.723
BERT-LSTM	0.739	0.803	0.73
PubMedBERT-LSTM	0.867	0.87	0.866

**Table 6:** Comparative results at the sentence level for the three types of candidates for the test corpus (positive and negative, and neutral classes)

Model name	Precision	Recall	F1
LogisticRegression	0.707	0.551	0.465
RandomForest	0.690	0.479	0.328
KNN	0.673	0.671	0.662
GaussianNB	0.496	0.471	0.335
DecisionTree	0.717	0.605	0.549
GradientBoosting	0.636	0.512	0.439
SVM (LCK)	0.373	0.587	0.456
SVM(Subtree Kernel)	0.404	0.345	0.372
NNB	0.623	0.637	0.63
CNN-LSTM	0.738	0.715	0.711
BERT-LSTM	0.826	0.824	0.824
PubMedBERT-LSTM	0.899	0.898	0.898

**Table 7:** K-Fold comparative results at the sentence level for identifying SNP-Phenotype associations with two classes of candidates (positive and negative-neutral classes)

Model name	Precision	Recall	F1
SVM	0.887	0.886	0.883
LogisticRegression	0.889	0.889	0.888
RandomForest	0.884	0.885	0.882
KNN	0.804	0.788	0.791
GaussianNB	0.834	0.809	0.814
DecisionTree	0.880	0.879	0.879
GradientBoosting	0.878	0.878	0.877
CNN-LSTM	0.895	0.895	0.895
BERT-LSTM	0.920	0.919	0.919
PubMedBERT-LSTM	0.925	0.925	0.924

**Table 8:** K-Fold comparative results at the sentence level for the three types of candidates (positive and negative, and neutral classes)

Model name	Precision	Recall	F1
SVM	0.866	0.865	0.853
LogisticRegression	0.874	0.877	0.873
RandomForest	0.868	0.873	0.867
KNN	0.805	0.812	0.802
GaussianNB	0.823	0.799	0.805
DecisionTree	0.863	0.862	0.861
GradientBoosting	0.870	0.869	0.868
CNN-LSTM	0.889	0.874	0.881
BERT-LSTM	0.906	0.901	0.902
PubMedBERT-LSTM	0.911	0.910	0.910

The results indicated that the pre-trained PubMedBERT LSTM model prepared by Microsoft performed better than other methods. BERT-LSTM and deep CNN-LSTM based models were ranked as the second-best performers among the studied methods. The presented results demonstrate that the higher performance of the three pre-mentioned methods is confirmed in both types of association extraction tasks with two and three classes.

It is worth noting that some experiments were conducted with the mentioned classifier at the abstract level (Table 9), whose performance was poorer than that of the same classifier at the sentence level. It can be concluded that although abstracts have more usable materials, some different related tasks must be efficiently employed for better performance. However, the Microsoft PubMedBERT-LSTM model, similar to the previous experiments, exhibited the best performance.

**Table 9:** Results for the association extraction at the abstract level for the test corpus with two classes of candidates (positive and negative-neutral classes)

Model name	Precision	Recall	F1
LogisticRegression	0.536	0.488	0.405
RandomForest	0.600	0.474	0.323
KNN	0.542	0.499	0.439
GaussianNB	0.626	0.504	0.400
BERT-LSTM	0.548	0.509	0.465
CNN-LSTM	0.537	0.477	0.345
PubMedBERT-LSTM	0.720	0.580	0.523

In addition to the mentioned experiments, we conducted several experiments to compare our results to the Deep-GDAE method developed by Nourani et al. [23]. For this purpose, we applied our PubMedBERT–LSTM method to GDAE and BeFree. Table 10 presents the results.

**Table 10:** Results of a comparison between our PubMedBERT-LSTM and the Deep-GDAE method

Test Corpus	PubMedBERT*–*LSTM based			Deep-GDAE [23]		
	Precision (%)	Recall (%)	F1-score (%)	Precision (%)	Recall (%)	F1-score (%)
GDAE	82.7	83.1	82.6	80.40	79.40	79.80
BeFree	75.7	80.6	77	66.00	73.80	69.60

As the table shows, our results outperformed the deep-FDAE method by 2.9% on GDAE and 7.4% on BeFree. We compared our results to the latest proposed method performed on the SNPPhenA corpus [27]. As Table 11 shows, our method surpassed the TreeLSTM method. Table 12 depicts the performance of the method proposed in [16] as well as our proposed PubMedBERT-LSTM. As the table shows, our method is comparable to the BioBERT base method.

**Table 11:** Results of a comparison between our PubMedBERT-LSTM and the TreeLSTM method

Test corpus	PubMedBERT–LSTM based			TreeLSTM [27]		
	Precision (%)	Recall (%)	F1-score (%)	Precision	Recall	F1-score
SNPPhenA	84	84.4	83.9	64.5	75.2	69.4

**Table 12:** Results of a comparison between our PubMedBERT-LSTM and the BioBERT based method

Test corpus	PubMedBERT–LSTM based			BioBERT based [36]		
	Precision (%)	Recall (%)	F1-score (%)	Precision (%)	Recall (%)	F1-score (%)
EUADR	78.1	83.6	80.7	75.03	76.17	79.97

**Fig. 12:**
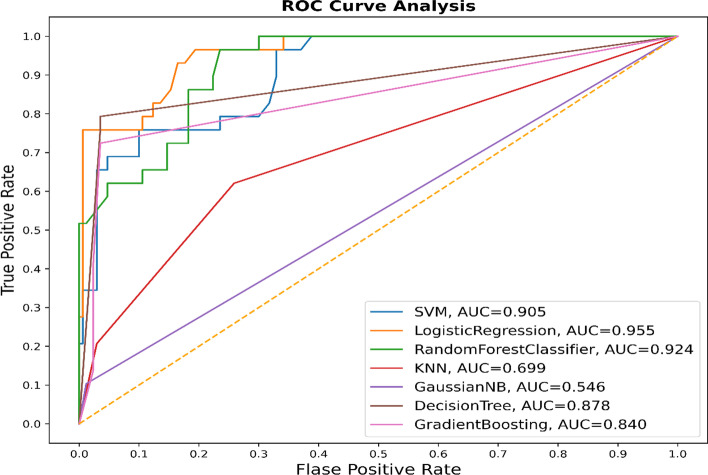
The ROC diagram of some of the used machine learning based methods for identifying SNP-Phenotype associations with two classes of candidates

As an indicator of the classifier's ability to distinguish between positive and negative classes, the AUC value measures the area under the curve. AUC value 1 is an indicator of a perfect classifier, and AUC value 0.5 indicates a classifier that does not perform better than random chance. In the Fig. 12, the logistic regression model has an AUC value of 0.955, which indicates that it is very effective at differentiating between the two classes. With an AUC value of 0.924, the random forest model performed well as well. In contrast, the Bayesian model had an AUC of only 0.546, indicating poor performance.

To verify the significance of the proposed methods, a sign test was conducted. The t-test is a statistical test used to determine whether two groups have significant differences in means. An analysis of the performance of different models was conducted using the t-test. In order to verify whether the results were random, a t-test was run once for both models and calculated. The p-value value was equal to 0.02 for two BERT-LSTM and CNN-LSTM models. Two BERT-LSTM models and PubMedBERT-LSTM models had p-value of 0.015. In addition, PubMedBERT-LSTM and CNN-LSTM models showed a p-value of 0.024. Based on the p-values, we can conclude that the differences between the models are not random.

### Forecasting degree of certainty

In addition to the experiments conducted to predict SNP-phenotype associations, the authors performed some experiments with machine learning and deep learning-based methods to identify the level of certainty of the associations. Tables 13, 14 and 15 present the comparative results of the algorithms for the test corpus and the k-fold results for the task. As the tables show, PubMed BERT-CNN LSTM performed better than other methods. However, the Deep CNN-LSTM-based method exhibited the next best performance.

**Table 13:** Results of the classification of the degree of certainty of associations at the sentence level for the test corpus using the different used methods

Model name	Precision	Recall	F1
LogisticRegression	0.730	0.741	0.729
KNN	0.692	0.741	0.716
GaussianNB	0.703	0.453	0.519
DecisionTree	0.784	0.700	0.719
GradientBoosting	0.761	0.771	0.761
MBS	81.4	51.9	63.4
BERT-LSTM	0.752	0.782	0.763
PubMedBERT-LSTM	0.782	0.8	0.773

**Table 14:** K-Fold results of the classification of the degree of certainty of associations at the sentence level with two classes of candidates (positive and negative-neutral classes)

Model name	Precision	Recall	F1
SVM	0.798	0.786	0.762
LogisticRegression	0.825	0.825	0.818
KNN	0.750	0.755	0.740
GaussianNB	0.788	0.758	0.765
DecisionTree	0.821	0.821	0.815
GradientBoosting	0.807	0.805	0.799
BERT	0.861	0.858	0.857
PubMedBERT + CNN	0.865	0.861	0.862
PubMedBERT + CNN LSTM	0.871	0.870	0.870

**Table 15:** K-Fold results of the classification of the degree of certainty of associations at the abstract level with two classes of candidates (positive and negative-neutral classes)

Model name	Precision	Recall	F1
SVM	0.766	0.752	0.730
LogisticRegression	0.774	0.777	0.770
KNN	0.744	0.745	0.733
GaussianNB	0.726	0.678	0.692
DecisionTree	0.787	0.790	0.781
GradientBoosting	0.772	0.775	0.765
Bert	0.821	0.817	0.814
PubMedBERT + CNN	0.826	0.820	0.822
PubMedBERT + CNN LSTM	0.834	0.835	0.831

In addition, we conducted some experiments using PubMed BERT + CNN and PubMedBERT + LSTM CNN methods to demonstrate the impact of the LSTM method. As the results in Tables 14 and 15 show, the use of LSTM improves the overall performance of the model.

## Discussion and conclusion

In this paper, several experiments were conducted with different machine and deep learning methods.

The experiments proved that the biomedically tuned pre-trained Bert-based models had the best performance compared to the other machine learning methods in the association extraction subtask. Additionally, the proposed rule-based method exhibited better performance than most of machine learning methods but poorer performance than deep learning-based methods. The uniform polarity of the sentences as well as the low proportion of complex sentences in the corpus could be influential factors in this regard.

The results also revealed that neutral candidates were important candidates to implement better relation extraction methods. Furthermore, the results demonstrated the importance of the degree of certainty of the association as a linguistic-based factor that could be used in addition to the existing methods to obtain more useful information.

The estimated degree of certainty of associations can be used, along with other factors such as abstract and paper confidence to define the overall degree of certainty and credibility of the extracted associations.

Although all existing relation extraction corpora and methods employ crisp relations, the authors maintain that it is an inefficient model for natural language relations, and they could be replaced with a better mathematical model called fuzzy relations (FR). Crisp relations deal with the binary relations between two entities in a sentence, while FRs include sets of fuzzy relations.

## Data Availability

Source code: https://github.com/mohamad-dehghani/SNPPhenA_XML. SNPPhenA Corpus: https://figshare.com/s/b18f7ff4ed8812e265e8.
